# Augmented Intelligence for Clinical Discovery in Hypertensive Disorders of Pregnancy Using Outlier Analysis

**DOI:** 10.7759/cureus.36909

**Published:** 2023-03-30

**Authors:** Ghayath Janoudi, Deshayne B Fell, Joel G Ray, Angel M Foster, Randy Giffen, Tammy J Clifford, Marc A Rodger, Graeme N Smith, Mark C Walker

**Affiliations:** 1 Epidemiology and Public Health, University of Ottawa, Ottawa, CAN; 2 Clinical Epidemiology, Ottawa Hospital Research Institute, Ottawa, CAN; 3 Maternal and Neonatal Research, Children's Hospital of Eastern Ontario, Ottawa, CAN; 4 Medicine, Health Policy Management and Evaluation, and Obstetrics and Gynecology, Saint Michael's Hospital, Toronto, CAN; 5 Health Sciences, University of Ottawa, Ottawa, CAN; 6 Medical Research, International Business Machines (IBM) Corporation, Ottawa, CAN; 7 Research, Canadian Institute of Health Research, Ottawa, CAN; 8 Medicine, McGill University, Montreal, CAN; 9 Obstetrics and Gynecology, Kingston General Hospital, Kingston, CAN; 10 Biomedical and Molecular Sciences, Queen’s University, Kingston, CAN; 11 Maternal and Nenonatal Research, University of Ottawa, Ottawa, CAN; 12 Obstetrics and Gynecology, University of Ottawa, Ottawa, CAN; 13 Obstetrics, Gynecology, and Newborn Care, The Ottawa Hospital, Ottawa, CAN; 14 Maternal and Nenonatal Research, Children’s Hospital of Eastern Ontario, Ottawa, CAN

**Keywords:** real-world data, clinical trials, research methods and design, augmented intelligence, hdp, preeclampsia treatment, clinical discovery, hypertensive disorders of pregnancy, preeclampsia-eclampsia

## Abstract

Objectives

Clinical discoveries are heralded by observing unique and unusual clinical cases. The effort of identifying such cases rests on the shoulders of busy clinicians. We assess the feasibility and applicability of an augmented intelligence framework to accelerate the rate of clinical discovery in preeclampsia and hypertensive disorders of pregnancy-an area that has seen little change in its clinical management.

Methods

We conducted a retrospective exploratory outlier analysis of participants enrolled in the folic acid clinical trial (FACT, N=2,301) and the Ottawa and Kingston birth cohort (OaK, N=8,085). We applied two outlier analysis methods: extreme misclassification contextual outlier and isolation forest point outlier. The extreme misclassification contextual outlier is based on a random forest predictive model for the outcome of preeclampsia in FACT and hypertensive disorder of pregnancy in OaK. We defined outliers in the extreme misclassification approach as mislabelled observations with a confidence level of more than 90%. Within the isolation forest approach, we defined outliers as observations with an average path length z score less or equal to -3, or more or equal to 3. Content experts reviewed the identified outliers and determined if they represented a potential novelty that could conceivably lead to a clinical discovery.

Results

In the FACT study, we identified 19 outliers using the isolation forest algorithm and 13 outliers using the random forest extreme misclassification approach. We determined that three (15.8%) and 10 (76.9%) were potential novelties, respectively. Out of 8,085 participants in the OaK study, we identified 172 outliers using the isolation forest algorithm and 98 outliers using the random forest extreme misclassification approach; four (2.3%) and 32 (32.7%), respectively, were potential novelties. Overall, the outlier analysis part of the augmented intelligence framework identified a total of 302 outliers. These were subsequently reviewed by content experts, representing the human part of the augmented intelligence framework. The clinical review determined that 49 of the 302 outliers represented potential novelties.

Conclusions

Augmented intelligence using extreme misclassification outlier analysis is a feasible and applicable approach for accelerating the rate of clinical discoveries. The use of an extreme misclassification contextual outlier analysis approach has resulted in a higher proportion of potential novelties than using the more traditional point outlier isolation forest approach. This finding was consistent in both the clinical trial and real-world cohort study data. Using augmented intelligence through outlier analysis has the potential to speed up the process of identifying potential clinical discoveries. This approach can be replicated across clinical disciplines and could exist within electronic medical records systems to automatically identify outliers within clinical notes to clinical experts.

## Introduction

Hypertensive disorders of pregnancy, including preeclampsia, are a leading cause of maternal and perinatal morbidity and mortality globally [1]. Despite considerable advancements in elucidating its pathophysiology, the management of preeclampsia has changed minimally over the past two decades [2-7]. Clinical trials of preeclampsia prevention have largely not shown positive results, except for aspirin in the prevention of early-onset preeclampsia [8-10].

Yet clinical observations play a pivotal role in the advancement of medical knowledge [11-13]. The description of an individual clinical case is sometimes the catalyzing step in generating new clinical research, by describing its aberrant or peculiar nature, and the formulation of a hypothesis of why this is so [12-18]. The latter was evident during the COVID-19 pandemic i.e., in understanding the natural history of COVID-19, its detection, and its clinical management [19-23].

Case reports and case series are the conventional study design used by clinicians and researchers to communicate a novel clinical discovery [16-18]. However, their recording and documentation depend on the busy clinician’s power of observation [24,25], and the prioritization by the journal editors who tend to offer case reports and case series low priority on the evidence-based medicine hierarchy of publication. Hence, case reports are now mostly used as educational tools [26-28].

Instead of relying on conventional and anecdotal case reports or case series, augmented intelligence uses a machine-based analytic approach to identify potentially new clinical discoveries and flag these to a human content-matter expert. The analytical part of augmented intelligence comprises any statistical or machine-learning method, including outlier analysis. Outlier analysis is currently used for financial fraud detection, network connection anomalies, malware detection, and manufacturing quality control [29,30]. It encompasses various statistical and machine-learning approaches that aim to identify observations that deviate significantly from the majority of other observations [29,30]. In health sciences research, outliers were conventionally handled as statistical noise and were excluded from analyses [31]. However, some outliers may arise from an unrealized and important mechanism or “signal” that holds valuable information and is, thus, worthy of further exploration [29,32].

Our study evaluated contextual outlier analysis using extreme misclassification to discover potentially new phenomena about preeclampsia and hypertensive disorders of pregnancy, using data from a completed randomized clinical trial and a separate cohort study. Extreme misclassification identifies observations that a predictive model mislabels with high confidence as outliers. In addition, the performance of the extreme misclassification contextual outlier analysis was contrasted with a more traditional point outliers detection algorithm to assess which can more aptly identify an outlier potentially useful for clinical discovery.

## Materials and methods

We conducted a retrospective exploratory outlier analysis of patients enrolled in the folic acid clinical trial (FACT) and the Ottawa and Kingston (OaK) cohort studies. The folic acid clinical trial was designed to capture preeclampsia diagnosis and progression and thus had a well-structured and focused dataset specific to preeclampsia. The OaK, as a prospective cohort study, represents the real world that aimed to capture myriad pregnancy complications, which may more accurately represent data gathered in usual clinical settings [8,33].

We used two methods within each dataset to identify outliers: isolation forest (a traditional point outliers approach) and extreme misclassification through a random forest predictive model (a contextual outlier approach).

As we are utilizing a predictive model development approach in our contextual outliers method (using the random forest outlier analysis approach), we followed the *transparent reporting of a multivariable prediction model for individual prognosis or diagnosis* (TRIPOD) statement for reporting i.e., transparent reporting of a multivariable prediction model for individual prognosis or diagnosis [34].

Data sources and description of participants

The FACT was a multi-national, double-blind, randomized, placebo-controlled trial completed in Argentina, Australia, Canada, Jamaica, and the UK. It aimed to assess the effects of a 4 mg daily folic acid supplementation on the development of preeclampsia in a high-risk obstetrics population. Between April 2011 and November 2015, a total of 2,464 participants were randomized in a 1:1 ratio to either folic acid or placebo. A total of 2,301 participants were included in the intent-to-treat analysis, with 14.1% of all participants developing new-onset preeclampsia [8].

The OaK was a prospective cohort study conducted from October 2002 to April 2009 that enrolled Canadian pregnant individuals at 12 to 20 weeks gestation who had a viable singleton or twin pregnancy. Data were systematically captured about maternal and infant demographics, maternal health, obstetrical history, and major pregnancy outcomes. One of these outcomes was an adjudicated outcome of hypertensive disorders of pregnancy. A total of 598 participants (7.4%) experienced a hypertensive disorder during pregnancy [33].

Study outcomes in FACT and OaK

The primary outcome of FACT, and that we used herein for the development of a predictive model, was the presence or absence of preeclampsia. Preeclampsia was defined as one of the following: 1) a diastolic blood pressure of ≥ 90 mm Hg on two occasions, four hours or more apart, and proteinuria (more than ++ on dipstick, 24-hour urinary protein ≥ 300 mg, or random protein:creatinine ratio ≥ 30 mg protein/mmol), each arising at 20+ weeks’ gestation; 2) development of the hemolysis, elevated liver enzymes, low platelets (HELLP) syndrome; or 3) superimposed preeclampsia namely, a history of pre-existing hypertension before 20 weeks gestation, with new-onset proteinuria at 20+ weeks [8,35].

The OaK cohort evaluated the adjudicated outcome of hypertensive disorders of pregnancy, which included chronic hypertension during pregnancy, pregnancy-induced hypertension, preeclampsia, and HELLP syndrome. The outcome was also used herein [33].

Features engineering

All FACT and OaK variables were used in our study with the exception of those meeting any of the following criteria: 1) a variation or a sub-categorization of the outcome; 2) a variable with over 50% missing data; 3) a variable used to re-categorize an existing continuous variable (e.g., age group when age was present); 4) a variable with only one unique value to all participants (e.g., death when no deaths occurred); 5) a variable where one unique value is present for 99% or more of all observations.

Considering our aim of identifying potential clinical discoveries through outlier analysis, we did not perform further data cleaning.

Sample size

As the sample sizes for FACT and OaK were fixed, no formal sample size calculation was completed for the current analyses. All 2,301 intent-to-treat participants in the FACT trial were included in our analyses, as well as all 8,085 participants from the OaK cohort [8,33].

Missing data

The overarching imputation strategy for missing data was that they were missing at random. We utilized an iterative imputation approach whereby missing data were imputed via a regression model by treating variables with missing data as a function of other variables. Iterative imputation performs this function in an iterated round-robin fashion in which a variable is designated as the dependent variable and the rest of the variables are designated as independent variables. This is done for every variable with missing data and repeated for a total of 10 iterations. The final imputation is the result of the tenth round [36-38].

We applied an exception to the previous imputation approach when it was clear that the missing data were due to a non-random mechanism. This includes missing data due to non-applicable questions i.e., those questions not posed to certain participants following a previous exclusionary question. This group of missing data cannot be assumed to be missing at random. In these cases, we introduced a numeric value to represent the lack of applicability for a continuous variable (for example, a zero in the case of a number of cigarettes), or a new category in the case of a categorical variable.

Outlier analysis methods

We analyzed each dataset for outliers using two approaches: the isolation forest, and extreme misclassification based on a random forest predictive model.

Isolation Forest

Isolation forest is an ensemble outlier detection recursive algorithm that works by assuming that the number of outliers is small and that they are easily isolated from the rest of the sample. This is conducted through a recursive process, whereby a variable is picked and portioned at random until all observations have been isolated individually. This process produces a decision tree structure in which the path length from the first split until the isolation of the observation can be measured. This process takes place across all variables until all observations have been isolated in all variables, at which point an average path across all variables is calculated for each observation [39]. We determined extreme outliers as observations with an average path length (also known as anomaly score) z score ≤ -3, or ≥ 3, using the programming language Python 3.9 (Python Software Foundation, Wilmington, DE, USA), together with NumPy, Pandas, and the Scikit-Learn software packages [40-43]. We tuned the isolation forest n_estimators and max_samples parameters by choosing the first value at which the mean and standard deviation of the anomaly score showed the least variations beyond that value, starting from a value of 100 n_estimator and 256 max_samples. For the contamination parameter, which provides the model with an assumption of the extent of outliers to be expected in the sample, it was assumed that no more than 5% of the sample could potentially be an outlier i.e., the contamination value was set at 0.05. Finally, we chose a random state value of 2022 namely, the year in which this analysis was conducted [40,43-45].

Random Forest

This second outlier approach required the development of a predictive model using the random forest algorithm and then leveraging the misclassified observations that the model wrongly predicted with a high level of confidence as an indicator of an outlier status (denoting extreme misclassification). The random forest algorithm is a widely used ensemble learning model in which several base estimators develop decision trees based on a random set of observations and a random set of variables, classifying the observation to the outcome of interest. The collective classification of all the base estimators forms the observation's final prediction [46]. To train, test, and validate the random forest model, the FACT dataset was split into a training set (with 1,200 observations), a testing set (with 800 observations), and a validation set (with 301 observations). Similarly, we split the OaK data into training, testing, and validation sets with 3,600, 2,400, and 2085 observations, respectively. We chose the parameters of the model through a random grid search approach, followed by a targeted grid approach that was informed by the results of the random grid search.

We considered those observations that the random forest model misclassified with a confidence level over 0.90 (90%) as outliers. The random forest confidence level represents the proportion of the base learners (or individual trees) that voted for the class. A threshold of 0.90 is an appropriately high threshold to allow for the capture of potentially novel observations whereby the possibility of an unmeasured but influential underlying mechanism could have been the cause of such misclassification.

We then constructed case narrative reports for the identified outliers by returning to the original datasets. Two content-expert researchers (GJ and MW) examined these case narratives and determined if an outlier had the potential of being a novel observation. We assessed each case narrative through several clinical considerations, as well as the clinical likelihood of experiencing the outcome given the available information. Specifically, the two reviewers asked three basic questions when reviewing each observation: 1) Is the presentation of the pregnant individual and the pregnancy journey likely to have led to the observed outcome?, 2) Are there any recorded potential effect modifiers that may have influenced the likely course of pregnancy and the resulting pregnancy outcome?, and 3) Is there any indication of potentially strong effect modifiers that are not recorded, such as an unrecorded medication or an undocumented chronic disease?

For a better understanding of the assessment process, see Table 1, where we present fictitious examples of case narrative assessments (actual case narratives are not publicly available to safeguard participants’ privacy and data).

**Table 1:** Fictitious example of clinical assessment of outliers BP: Blood pressure; LFT: Liver function test; PE: Pulmonary embolism; SSRIs: Selective serotonin reuptake inhibitors

ID	Assessment	Reasoning	Risk Factors	Interesting Variables
123-456	Potential novelty	The participant had PE, BP measured at 187/103 mm HG, elevated LFT, and required delivery at 34 weeks gestation. The number and type of risk factors do not necessarily justify this presentation. Also, the participant suffers from depression and indicated concomitant medication. Could the concomitant medication predispose the participant to the observed outcome? This would be worth further examination.	Overweight (BMI 29), new partner	SSRIs in concomitant medication, no perinatal vitamins
123-678	Natural deviation	The participant exhibited PE with BP at 140/90 mm HG and 3+ urine dipstick. The participant delivered at 39 weeks gestation with no complications. Considering existing risk factors and positive overall maternal and fetal outcomes, this clinical scenario is not unexpected.	Previous history of PE, obesity (BMI 33), advanced maternal age	Aspirin, folic acid, calcium supplements

Based on the clinical review (completed by GJ and MW), we classified each observation as either a “potential novelty” or a “natural deviation.” We also attempted to explain why either type of observation was captured as an outlier in its given model. Based on this assessment, we reported the proportion of potential novelties detected by each algorithm within each dataset.

## Results

Characteristics of participants in FACT and OaK

Participants in both studies were of similar age and shared similar prior pregnancy characteristics such as gravidity and multiple pregnancies. Participants in the FACT study had more risk factors for preeclampsia than those enrolled in OaK, including a higher mean weight (91.6 versus 70.4 kg), a higher proportion of multiple pregnancies (18.6% versus 1.4%), past hypertension (18.4% versus. 1.3%), and a history of preeclampsia (25.3 versus 2.9%), respectively (Table 2).

**Table 2:** Maternal and medical characteristics of the FACT and OaK participants FACT: Folic acid clinical trial; NA: Not applicable; OaK: Ottawa and Kingston birth cohort; SD: Standard deviation

Variable	FACT (N=2,301)	OaK (N=8,085)
Age in years, mean (SD)	31.4 (5.3)	30.3 (5.3)
Weight in kg, mean (SD)	91.6 (24.8)	68.7 (19.9)
Height in cm, mean (SD)	NA	163.7 (14.4)
Body mass index in kg/m^2^, mean (SD)	34.0 (11.2)	NA
G (gravidity), median (interquartile range)	2 (2 to 4)	2 (1 to 3)
T (number of term births), median (interquartile range)	1 (0 to 1)	1 (0 to 1)
P (number of preterm births), median (interquartile range)	0 (0 to 0)	0 (0 to 0)
A (number of abortions/miscarriage), median (interquartile range)	0 (0 to 1)	0 (0 to 2)
L (number of living children), median (interquartile range)	1 (0 to 1)	1 (0 to 1)
M (number of multiple pregnancies), median (interquartile range)	0 (0 to 0)	0 (0 to 0)
Current multiple pregnancy, n (%)	428 (18.6)	361 (4.5)
Assisted reproductive technology pregnancy, n (%)	213 (9.3)	359 (4.4)
History of chronic hypertension, n (%)	428 (18.6)	101 (1.3)
History of gestational diabetes, n (%)	NA	141 (1.7)
History of diabetes mellitus, n(%)	311 (13.5)	NA
History of preeclampsia, n (%)	581 (25.3)	237 (2.9)
History of smoking, n (%)	919 (39.9)	766 (9.5)
Smoking during pregnancy, n (%)	173 (7.5)	NA
Alcohol use during pregnancy, n (%)	48 (2.1)	NA
Folic acid supplementation (not intervention related), n (%)	1,882 (81.8)	1,786 (22.1)
Aspirin, n (%)	665 (28.9)	NA
Calcium supplementation, n (%)	196 (8.5)	NA
Other medications, n (%)	1,620 (70.4)	2,740 (33.9)

There were no missing data for the outcomes assessed in the random forest model in either dataset. The FACT study contributed a total of 84 variables in both the random forest and isolation forest models; of these, 34 variables had missing values. The OaK cohort contributed a total of 72 variables to the random forest and isolation forest models; of these, 48 variables had missing values. Missing data were handled according to the missing data imputation plan described previously.

Model development, specification, and performance

Isolation Forest

Tuning the isolation forest model produced the following parameters for FACT (N=2,301) data: n_estimator = 300, max_samples = 700. And it produced the following parameters for OaK (N=8,085) data: n_estimator = 900 and max_samples = 256. As per our methods, we used the value of 2,022 as our random_state and 0.05 for contamination. The remaining tuning parameters were left at default values (bootstrap = False, max_features = 1.0, n_jobs = None, warm_start = False). The model generated an anomaly score for each observation, which we used as the basis to determine outliers.

As the isolation forest is an unsupervised model, outcome-based model performance measures could not be calculated.

Random Forest

The FACT dataset included 2,301 participants, 325 (14.1%) of who experienced the outcome of preeclampsia. The OaK dataset included 8,085 participants; 597 (7.4%) experienced the outcome of hypertensive disorders during pregnancy. Tuning the FACT model resulted in the following parameters: bootstrap = False, max_depth = 10, max_features = 40, min_samples_leaf = 2, min_samples_split = 2, and n_estimators = 600. Tuning the model for OaK resulted in the following parameters: n_estimators = 100, min_samples_split = 6, min_samples_leaf = 1, max_features = 10, max_depth = 90, and bootstrap = False.

Using the validation dataset, the FACT random forest model showed a precision of 0.97 and a recall of 0.83 for the outcome of preeclampsia. The OaK random forest model showed a precision of 0.75 and a recall of 0.08 for the outcome of hypertensive disorders of pregnancy. The performance of the models over the entirety of the FACT and OaK datasets is displayed in Table 3.

**Table 3:** Classification report metrics for random forest model applied on the full dataset (training, test, and validation sets) FACT: Folic acid clinical trial; OaK: Ottawa and Kingston birth cohort * The FACT outcome was preeclampsia or hemolysis, elevated liver enzymes, low platelets (HELLP) syndrome. The OaK outcome was a hypertensive disorder of pregnancy.

Class	FACT Precision	FACT Recall	OaK Precision	OaK Recall
No outcome* present	0.99	0.99	0.96	1.00
Outcome* present	0.95	0.93	0.97	0.50

Description of outliers

Isolation Forest

Based on setting outliers at a z score of ≤ -3 or ≥ 3, 19 outliers (0.8%) were identified in the FACT dataset and 172 outliers (2. 1%) were identified in the OaK dataset.

Outliers in each of the datasets display different sets of participants’ baseline and demographic characteristics that set them apart from their original datasets. These characteristics are outlined in Table 4.

**Table 4:** Characteristics of isolation forest and random forest outliers and their original datasets FACT: Folic acid clinical trial; NA: Not applicable; OaK: Ottawa and Kingston birth cohort; SD: Standard deviation * Cells with medical information pertaining to less than seven aggregate participants are suppressed to protect privacy, and safeguard participants' data.

Variable	FACT Isolation Forest Outliers (N = 19)	FACT Random Forest Outliers (N = 13)	Original FACT Dataset (N = 2,301)	OaK Isolation Forest Outliers (N = 172)	OaK Random Forest Outliers (N = 98)	Original OaK Dataset (N = 8,085)
Age in years, mean (SD)	30.1 (5.2)	29.8 (4.6)	31.4 (5.3)	25.7 (10.2)	30.4 (5.7)	30.3 (5.3)
Weight in kg, mean (SD)	96.9 (28.6)	82.3 (22.8)	91.6 (24.8)	60.1 (30.2)	69.4 (22.8)	68.7 (19.9)
Height in cm, mean (SD)	NA	NA	NA	139.2 (59.3)	163.6 (17.3)	163.7 (14.4)
BMI in kg/m^2^, mean (SD)	35.6 (9.4)	31.0 (8.6)	34.0 (11.2)	NA	NA	NA
G (gravidity), median (interquartile range)	3 (2 to 4.5)	2 (2 to 3)	2 (2 to 4)	2.8 (2 to 4)	2 (1 to 3)	2 (1 to 3)
T (number of term births), median (interquartile range)	1 (0.5 to 1.5)	1 (0 to 1)	1 (0 to 1)	2 (1 to 2)	0 (0 to 1)	1 (0 to 1)
P (number of preterm births), median (interquartile range)	0 (0 to 1)	0 (0 to 1)	0 (0 to 0)	2 (0 to 2)	0 (0 to 0)	0 (0 to 0)
A (number of abortions/ miscarriage), median (interquartile range)	1 (0 to 1)	0 (0 to 1)	0 (0 to 1)	2 (1 to 2)	0 (0 to 1)	0 (0 to 2)
L (number of living children), median (interquartile range)	2 (1 to 2)	0 (0 to 1)	1 (0 to 1)	2 (1 to 2)	0 (0 to 1)	1 (0 to 1)
M (number of multiple pregnancies), median (interquartile range)	0 (0 – 0)	0 (0 – 0)	0 (0 – 0)	2 (0 – 2)	0 (0 – 0)	0 (0 – 0)
Current multiple pregnancy, n (%)	7 (36.8)	Suppressed*	428 (18.6)	141 (82.0)	Suppressed*	361 (4.5)
Assisted reproductive technology pregnancy, n (%)	Suppressed*	Suppressed*	213 (9.3)	Suppressed*	Suppressed*	359 (4.4)
History of chronic hypertension, n (%)	7 (36.8)	Suppressed*	423 (18.4)	Suppressed*	0 (0)	101 (1.3)
History of gestational diabetes, n (%)	NA	NA	NA	Suppressed*	Suppressed*	141 (1.7)
History of diabetes mellitus, n(%)	6 (31.6)	Suppressed*	311 (13.5)	NA	NA	NA
History of pre-eclampsia, n (%)	10 (52.6)	Suppressed*	581 (25.3)	7 (4.1)	Suppressed*	237 (2.9)
History of smoking, n (%)	13 (68.4)	Suppressed*	919 (39.9)	36 (20.9)	9 (9.2)	766 (9.5)
Smoking during pregnancy, n (%)	13 (68.4)	Suppressed*	173 (7.5)	NA	NA	NA
Alcohol use during pregnancy, n (%)	0 (0)	Suppressed*	48 (2.1)	NA	NA	NA
Folic acid supplementation, n (%)	9 (47.4)	11 (84.6)	1,882 (81.8)	43 (25.0)	22 (22.4)	1,786 (22.1)
Aspirin, n (%)	Suppressed*	Suppressed*	665 (28.9)	NA	NA	NA
Calcium supplementation, n (%)	Suppressed*	Suppressed*	196 (8.5)	NA	NA	NA
Other medications, n (%)	14 (73.7)	10 (76.9)	1,620 (70.4)	59 (34.3)	34 (34.7)	2,740 (33.9)
Last systolic blood pressure measurement during pregnancy, mean (SD)	101.9 (72.5)	142.8 (17.3)	136.1 (18.2)	NA	NA	NA
Last diastolic blood pressure measurement during pregnancy, mean (SD)	61.9 (43.3)	82.9 (10.5)	81.5 (13.0)	NA	NA	NA
Gestational age at delivery, mean (SD)	32.8 (5.9)	38.3 (1.9)	37.8 (2.8)	9.9 (7.7)	38.5 (4.4)	38.0 (6.0)
Individuals with pre-eclampsia, n (%)	8 (42.1)	12 (92.3)	325 (14.1)	Suppressed*	33 (33.7)	249 (3.1)
Individuals with hypertensive disorders of pregnancy, n (%)	NA	NA	NA	Suppressed*	98 (100.0)	597 (7.4)

We noticed that FACT outliers, compared to the original full dataset, had similar mean age, mean weight, and mean BMI; there was a similarity in the proportion of individuals reporting alcohol use during pregnancy, and the proportion of individuals reporting the use of concomitant medications was also similar. Beyond that, outliers had a higher number of previous pregnancies, more twin pregnancies, and a higher proportion of participants with a history of chronic hypertension, diabetes, eclampsia, and smoking. The FACT outliers were less likely to be on folic acid supplementation or aspirin and had their delivery at a lower weeks gestation age than the full dataset. The FACT outliers also had lower mean systolic and diastolic measures than the original dataset, but that also came with a larger standard deviation than observed in the original dataset. All of these observations indicate that FACT outliers appear to be at a higher risk of developing preeclampsia, which is reinforced by higher proportions of FACT outliers experiencing preeclampsia than in the original dataset.

The OaK outliers had similar proportions of individuals with a history of chronic hypertension, gestational diabetes, and history of preeclampsia. Also, they were similarly likely to have been on folic acid supplementation or other concomitant medication. They were less likely to develop preeclampsia or hypertensive disorders during pregnancy. The OaK outliers displayed a markedly lower gestational age at delivery than the original dataset.

We also observed that the outliers identified through the isolation forest had a high proportion of lost-to-follow-up cases. Specifically, there were 12 outliers in the FACT outliers that were lost to follow-up (63.2%) compared to an overall 11.3% in the original full dataset. Similarly, there were 85 outliers in the OaK outliers that were lost to follow-up (49.4%) compared to 2.3% overall lost-to-follow-up in the original full dataset.

Random Forest

Under the random forest model, 13 outliers (0.6%) were identified in the FACT dataset and 98 outliers (1.2%) in the OaK dataset. We have presented the characteristics of these outliers in contrast to the original datasets in Table 4.

In both datasets, the outliers (identified through the random forest extreme misclassification approach) were of similar age; gravidity, term, pre-term, abortion, living (GTPAL); history of chronic hypertension; folic acid supplementation (outside of FACT intervention); and gestational age at delivery as in their original datasets.

The FACT outliers also had a similar proportion of individuals with a history of diabetes, current aspirin intake, and mean diastolic blood pressure as in the full FACT dataset. The FACT outliers had lower weight and BMI, fewer individuals with multiple pregnancies, fewer pregnancies with assisted reproductive technology, and fewer individuals with a history of smoking. They also had a higher proportion of individuals with a history of preeclampsia, smoking during pregnancy, the use of alcohol during pregnancy, the use of calcium channel blockers, and the use of other medications. Moreover, FACT outliers had higher mean diastolic blood pressure than the original full dataset. The OaK outliers had similar weight, height, history of gestational diabetes, history of preeclampsia, and proportion of participants taking medications.

Under the extreme misclassification approach, FACT and OaK outliers display a much higher proportion of participants with preeclampsia and hypertensive disorders of pregnancy than their full datasets. We also observed that while lost-to-follow-up cases were higher in FACT outliers at two cases (15.4%) than the original dataset (11.3%), this was considerably lower than the proportion of lost-to-follow-up cases in FACT outliers identified through the isolation forest. There were no lost-to-follow-up cases in the OaK outliers.

Assessment of outliers

**Figure 1:**
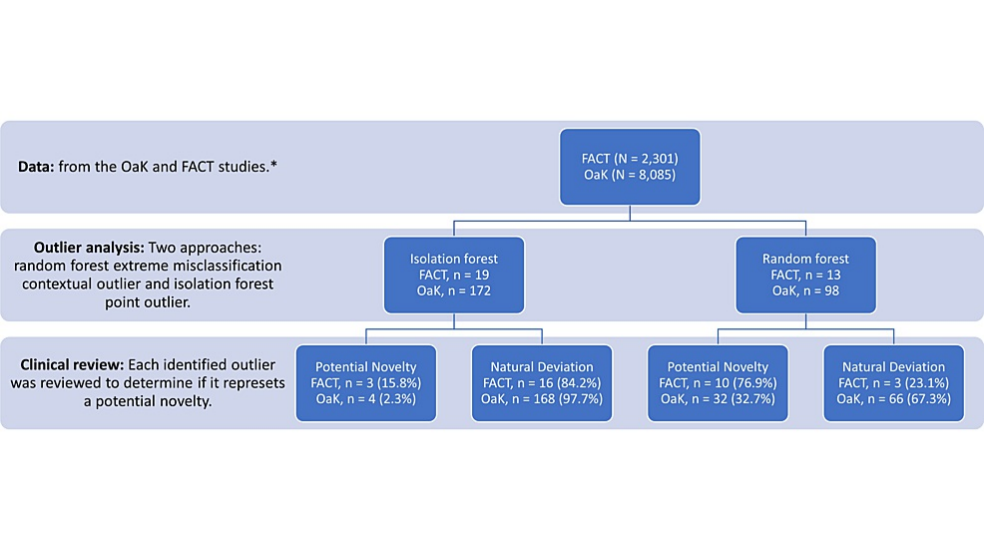
Flowchart of results per analysis stage * Data from each study were analyzed separately and were not combined

Upon review of each outlier observation identified in the FACT dataset, we determined that three outliers (15.8%) in the isolation forest and 10 (76.9%) in the random forest extreme misclassification approaches were potential novelties worthy of further investigation. Similarly, upon review of each outlier observation identified in the OaK dataset, we determined that four outliers (2.3%) in the isolation forest and 32 (32.7%) in the random forest extreme misclassification approaches were potential novelties worthy of further investigation. A summary of our assessment can be found in Table 5. Our findings are further outlined as a flowchart in Figure 1.

**Table 5:** Detected outliers and results of the assessment of their case narratives FACT: Folic acid clinical trial; OaK: Ottawa and Kingston birth cohort

Model	FACT	OaK
Isolation forest		
Total outliers identified, n	19	172
Outliers determined as potential novelty, n (%)	3 (15.8)	4 (2.3)
Outliers determined as natural deviation, n (%)	16 (84.2)	168 (97.7)
Random forest extreme misclassification		
Total outliers identified, n	13	98
Outliers determined as potential novelty, n (%)	10 (76.9)	32 (32.7)
Outliers determined as natural deviation, n (%)	3 (23.1)	66 (67.3)

## Discussion

In this study, we demonstrated the feasibility of applying an augmented intelligence outlier analysis framework on clinical trials and real-world data. This approach could accelerate the rate of clinical discovery that has traditionally depended on the observation and research skills of individual clinicians. Although the use of outlier analysis for uncovering new clinical insights was first proposed in a publication in 2000 by Laurikkala et al. [47], to the best of our efforts we were unable to find published studies approaching this problem from a contextual outlier analysis perspective using extreme misclassification of a random forest model.

Out of 2,301 participants in the FACT study, we identified 19 outliers using the isolation forest algorithm and 13 outliers using the random forest extreme misclassification approach. Of these, a clinical review determined that three (15.8%) and 10 (76.9%) were potential novelties in the isolation forest and random forest extreme misclassification approaches, respectively, warranting further investigation through source documents review and participant follow-up. Out of 8,085 participants in the OaK study, we identified 172 outliers using the isolation forest algorithm and 98 outliers using the random forest extreme misclassification approach; four (2.3%) and 32 (32.7%), respectively, were potential novelties worthy of further investigation.

In both FACT and OaK datasets, there were more potential novelties within the outliers identified through the random forest extreme misclassification approach (FACT 76.9%, OaK 32.7%) than through the isolation forest approach (FACT 15.8%, OaK 2.3%). Several observations can be made from these results. First, our contextual outlier analysis approach using extreme misclassification has captured a higher number and proportion of potential novelties compared to a standard outlier approach using isolation forest in both datasets. This indicates the advantage of the contextual outlier approach using extreme misclassification. Despite capturing a lesser total number of outliers, it still managed to produce a higher number of potential novelties. This can also be explained by the tendency of the isolation forest algorithm to identify observations that are the easiest to isolate, which manifested in capturing cases that were lost-to-follow-up, withdrew from the study, or experienced early termination. These cases had little information to allow the clinical review to determine if there were any potential novelties.

A second observation was that regardless of the model, we noticed an overall higher proportion of potential novelties in FACT outliers than in OaK outliers. This may be due to the structure and type of variables (features) of the underlying data. For example, the FACT study collected several blood pressure measurements as well as laboratory tests throughout the trial. In contrast, no similar longitudinal variables were collected in the OaK study. This observation is likely to inform future expectations of the proportion of outliers and novelties within datasets based on the dataset design and structure. It is important to note that only one observation within the OaK dataset was identified by both outlier methods; the remaining were unique to each approach. Similarly, all outlier observations within the FACT dataset were unique to each approach with no overlap. This could be explained by the inherently different assumptions and definitions of outliers in each model, whereby the isolation forest depends on unique values in the features. In contrast, the extreme misclassification approach depends on the poor fit of an observation to an outcome classification model.

Based on the results obtained in this study, we believe that the extreme misclassification approach is likely to perform better on both real-world data and clinical trial data. However, considering our previous observation of the minimum overlap in outliers between the two approaches, it is possible that there is room for both approaches to work in parallel to maximize the number of identified potential novelties.

Across all of the datasets and approaches, our clinical review identified 49 observations as potential novelties based on the case narratives of these observations. Ideally, the next step would be to investigate source documentation and potentially follow up with these participants to understand better if an unrecorded factor may have contributed to the unusual clinical observations. Based on the unstructured notes that were added throughout the clinical review of each outlier, there seems to be a common theme supporting further investigation of the potential role of recorded and unrecorded concomitant medications in contributing to the nature of the observations. Moreover, another theme emerged that indicated that there is room to reassess the traditional importance of certain risk factors when they are simultaneously present with concomitant medication. Both themes can serve as the basis for investigating novelties and formulating scientific hypotheses.

There were several limitations in our study. These include the lack of blinding of the clinical reviewers to the type of model that identified the outlier being assessed. This could have biased the determination of potential novelties in favor of the new approach of extreme misclassification. Blinding the clinical reviewers was not feasible as they needed the model information insight to better understand why a given observation is identified as an outlier. Another important limitation was the lack of a final assessment of the performance of these outlier models in identifying true novel cases that could lead to a clinical discovery. This final assessment can only occur after the determined potential novelties are investigated further through source document review and participants’ follow-up, which we were unable to do in this study. Another potential limitation was that the actual outcome for the classification model in both datasets was relatively low (14.1% in FACT and 7.4% in OaK), which may have caused an imbalanced classification, leading to poor recall of the random forest model in both datasets. Within the extreme misclassification approach, the poor recall meant that there was a higher proportion of outliers that were misclassified as not having the outcome compared to outliers that were misclassified as having the outcome. It is also important to note that the classification model is not meant to act as a clinical prediction model and included variables such as the fetal Apgar score, gestational age at delivery, blood pressure measurements, and laboratory results that would not be useful for the development of a clinical prediction model. Finally, it is important to note that the generalizability of the finding is that extreme misclassification performs better than isolation forest and may be limited considering the use of two studies, both of which are in the field of obstetrics.

## Conclusions

Unique and unusual clinical observations have been the catalyst for clinical discovery. An efficient way is needed to identify and pursue such observations. In this study, we demonstrated the feasibility, applicability, and potential benefits of utilizing augmented intelligence outlier analysis methods to accelerate the rate of clinical discovery, particularly in preeclampsia and hypertensive disorders of pregnancy. Furthermore, we applied our proposed extreme misclassification contextual outlier analysis approach to real-world and clinical trial data by using the datasets from the FACT and OaK studies as test cases. Our results have shown a higher proportion of potential novelties under the extreme misclassification approach compared to the isolation forest approach in both the clinical trial and real-world data. Our findings suggest that an extreme misclassification contextual outlier approach may have advantages over the classical point outlier analysis approach in identifying cases with potential novelty and thus accelerating the rate of clinical discovery. The application of augmented intelligence using outlier analysis to accelerate the rate of clinical discovery can be implemented in various clinical disciplines and utilized within electronic medical records to identify outliers to clinical experts automatically.
